# Public preferences for government spending in Canada

**DOI:** 10.1186/1475-9276-11-64

**Published:** 2012-10-30

**Authors:** Sabrina Ramji, Carlos Quiñonez

**Affiliations:** 1 Community Dental Health Services Research Unit, Faculty of Dentistry, University of Toronto, 124 Edward Street, Toronto, ON, M5G 1G6, Canada

**Keywords:** Public preferences, Health care, Priority setting, Health services needs and demand

## Abstract

This study considers three questions: 1. What are the Canadian public’s prioritization preferences for new government spending on a range of public health-related goods outside the scope of the country’s national system of health insurance? 2. How homogenous or heterogeneous is the Canadian public in terms of these preferences? 3. What factors are predictive of the Canadian public’s preferences for new government spending? Data were collected in 2008 from a national random sample of Canadian adults through a telephone interview survey (n =1,005). Respondents were asked to rank five spending priorities in terms of their preference for new government spending. Bivariate and multivariable logistic regression analyses were conducted. As a first priority, Canadian adults prefer spending on child care (26.2%), followed by pharmacare (23.1%), dental care (20.8%), home care (17.2%), and vision care (12.7%). Sociodemographic characteristics predict spending preferences, based on the social position and needs of respondents. Policy leaders need to give fair consideration to public preferences in priority setting approaches in order to ensure that public health-related goods are distributed in a manner that best suits population needs.

## Introduction

Canada is well known for its publicly funded, universal system of health insurance (Medicare), instituted to provide all citizens with access to medically necessary hospital and physician services on the basis of need rather than the ability to pay. Despite this, evidence points to persisting socio-economic inequity in healthcare use. This is in part due to individual provinces holding the responsibility for planning and funding the delivery of their own health services, resulting in variation in health system characteristics across the country and subsequently, different degrees of inequity in access [1]. Furthermore, many services that Canadians rely upon fall beyond the scope of Medicare, preventing them from achieving comprehensive access to a broad range of health and social services. In turn, a strong role exists in Canada for private out-of-pocket and employer-based insurance arrangements for many socially justifiable services. In this regard, approximately 30% of total health care costs in Canada are covered privately, including pharmaceuticals and dental care [2]. Similarly, certain social services such as child care, which have an integral impact on health, do not receive strong public subsidies [3-5].

In Canada, as in many other international contexts, there are widespread disparities in access to and utilization of non-insured health-related goods and services. Access to these goods and services has been found to be strongly related to an individual's income and/or the presence of private health insurance [6]. In particular, the experience of the working poor and other low-income Canadians is problematic as individuals who are in low-wage employment arrangements have less access to employment-based benefits (such as dental insurance, prescription drugs and vision care) than do higher-wage workers, and in cases such as dental care, may even have less access to care when compared to those with no incomes who often benefit from public insurance coverage [7-9]. Research has also shown that individuals with below average incomes are less likely to have filled a prescription due to financial constraints compared to those with above average incomes, and are more likely to not visit a dentist due to costs [10,11]. Similar segments of the population are vulnerable to access barriers in countries such as United States, Australia and New Zealand where such services are also heavily dependent on income. In Canada, disparities in access for these non-insured health-related goods and services are occurring in the context of a largely publicly funded health care system and are of concern due to such things as untreated morbidity and potential economic impacts on the health care system and society at large [12].

In countries such as Canada, where there is publicly funded health care, resources are limited and therefore cannot meet the health needs of all, necessitating the setting of priorities. Priority setting is an extremely complex task as it requires choosing between competing values for resources [13,14]. Daniels and Sabin (2008) propose meeting health care needs under resource constraints fairly by limiting resources in a publicly accountable way. They argue that the public systems should engage the public on these limits as a way of ensuring the health care system reflects the values for which it is designed. They reason that such public engagement (or similarly public involvement or participation) may not lead to consensus or unanimity but will contribute to the attainment of legitimacy, since the reasons advanced in support of a particular position are those which the general public can accept as relevant and appropriate given resource constraints [15,16].

Dhalla et al. recently considered options for broadening the funding base for health care in Canada [17]. They concluded that revenue could be raised through general taxation by eliminating the private health insurance subsidy available in all provinces except Quebec (which does not tax non-wage benefits) and/or by raising funds through social insurance or sin taxes (revenues garnered from the purchase of potentially harmful and indulgent resources) [18] Yet the question remains as to whether an expanded funding base would be used only to maintain current growth, or also to expand current service levels. With recent federal government elections and upcoming provincial government elections coupled with a renegotiation of the federal-provincial health accord in 2014 (which periodically revisits agenda-setting in health care), the issue of potential expansions to the Medicare envelope is highly relevant. It is known that the Canadian public is in support of government spending for currently unfunded health services [19]. Thus, this study is an attempt to consider this issue by enumerating Canadian preferences for new government spending on a range of public services currently outside the scope of Medicare.

While previous Canadian surveys have attempted to determine public preferences for government spending within the health care domain, items included in the priority lists have generally been those within the scope of Medicare [20-22]. Knowledge of how the Canadian public prioritizes government spending for those public health-related goods currently outside the scope of the national health insurance system has not been explored. This study is the first, to our knowledge, to examine public preferences for new government spending on prioritization of a wide range of public health goods currently outside the scope of Medicare (i.e. home care, pharmacare, dental care, vision care and child care). These spending priority areas have been found to be repeatedly requested by Canadian citizens for improved government support, and are the largest expenditures for uninsured health and social services in Canada [17,19]. In particular, child care, a non-traditional health service, is included in our priority list, as well as dental care, a highly demanded service and commonly neglected item in health care priority discussions. Until now, studies have only explored public preferences for government spending on home care, pharmacare and child care independently. This study aims to ascertain public preferences for the aforementioned services, in relation to each other. Exploring public preferences can inform new government spending generally, and by comparing current methods of financing with public preferences, can specifically show if there are differences between government approaches and public priorities. Examining the social and economic circumstances underlying preferences can also help identify specific populations to whom policies and/or programs could be tailored [23].

This paper will consider three questions: 1. What are the Canadian public’s preferences for new government spending on a range of public health-related goods outside the scope of Medicare? 2. How homogenous or heterogeneous is the Canadian public in terms of these preferences? 3. What sociodemographic factors are predictive of the Canadian public’s preferences for new government spending?

## Methods

Data were collected from a provincially stratified sample of 1,005 Canadians aged 18 years and over. A sample size calculation indicated that this could provide a 3.0% margin of error with 95% confidence relative to the Canadian population. The survey was conducted by telephone interview in February 2008. A market research firm was employed to administer the survey as part of their weekly national omnibus survey. Participants were selected using random digit dialing. Calls were made from Wednesday to Sunday, between 4:30pm and 9:00pm on weeknights and 10:00am to 8:00pm on weekend days, respondent’s time. Approximately 48,000 calls were attempted, and excluding numbers that were not in service, fax machines, or invalid, there were approximately 37,000 eligible calls. When excluding busy signals, answering machines, no answer, language barrier, ill or incapable respondents and eligible persons not available, a total of approximately 11,000 people were asked to participate. A response rate of approximately 3.0% was achieved. Willingness to participate was taken to imply consent, and no personal identifiers were collected. Surveys were conducted in English or French. The study received approval by the University of Toronto’s Office of Research Ethics.

The outcome variables were based on one survey question: “If the government were to provide the money, which of the following services would be most important to you?” The five spending priorities were randomized and thus read in no particular order and included (1) pharmacare; (2) dental care; (3) child care; (4) home care; and (5) vision care. There was no option to select the choice of no government spending. After the first choice was taken, the respondent was then asked: “Which of the following remaining services would be second most important to you?” They were read the remaining choices in the order that they were initially presented. This continued with respondents choosing their third and fourth choice, with the fifth choice documented by default*.* For any spending priority, respondents ranking the service as their top priority were assigned a value of “1”; respondents ranking all other services as their top priority were assigned “0”. This dichotomization created the following outcome variables: pharmacare spending priority, dental care spending priority, child care spending priority, home care spending priority, and vision care spending priority.

The main independent and predictive variables were sex, age, household income, education, private health insurance coverage, parenthood, and residence status. These variables are consistent with prior research on public opinion and preferences concerning the welfare state [24-28]. Reported age at the time of the survey was grouped into four categories: 18 to 29, 30 to 44, 45 to 64, 65 years or older. Socioeconomic status (SES) was measured by total household income and the highest level of education attained at the time of the survey. Total household income was categorized into five groups: less than $20,000, $20,000 to less than $40,000, $40,000 to less than $60,000, $60,000 to less than $80,000 and $80,000 or higher. Highest educational level was categorized as having high school diploma or less, having attended college or having a university and/or post-graduate degree. Private health insurance status was dichotomized into insured or uninsured based on respondent’s predominant mode of payment. Individuals were classified as insured if they reported dental care to be covered by private health insurance or public assistance program. Individuals were classified as having no health (dental) insurance coverage if they reported out-of-pocket payments for dental care. The role of parent and caregiver of children was assumed if the respondent lived in a household with children under 6 years of age and comparison was made with those who had no children under 6 years of age in the household (includes respondents who have no children in the household or those who have children 6 years of age or older. A variable for residence status indicated whether respondents’ residence was urban or rural. This was defined by Statistic Canada’s notion of census metropolitan area: an area was urban if the census subdivision’s population was at least 100,000 at the previous census [29].

Simple descriptive statistics were generated for the sample as a whole and for the five subgroups based on the predictive variables. Bivariate logistic regression analysis was conducted to assess associations between outcome variables and each of the predictive variables. Multivariable regression models were built for each spending priority to identify dominant predictors and to control for potential confounding. Independent variables were removed from the models if they did not show an independent effect of p<0.05 on preferences at the bivariate level and if they did not change the parameters of the remaining variables by 20% or greater with their removal. An analysis for vision care was excluded as nothing was found to be predictive of spending for this service, likely due to the small number preferring this service. All estimates and statistics were computed using a statistical software package (SPSS). Descriptive estimates are weighted by age and sex, according to 2006 census data to reflect the Canadian population.

## Results

As a first priority, this sample of Canadian adults prefers spending on child care (26.2%), followed by pharmacare (23.1%), dental care (20.8%), home care (17.2%), and vision care (12.7%). Preferences for new government spending vary according to sociodemographic characteristics. Table 1 presents the weighted percentages of preferences by these characteristics.

**Table 1:** Socio demographic characteristics of Canadian adults prioritizing new government spending

**Characteristics**	**First priority ranking for new government spending**				
	**Child care (%)**	**Pharmacare (%)**	**Dental care (%)**	**Home care (%)**	**Vision care (%)**
**Age**					
18–29	34.7	11.3	22.1	10.6	20.8
30–44	38.6	25.9	26.3	21.3	29.1
45–64	23.1	47.2	37.9	40.6	36.3
65+	3.6	15.6	13.7	27.5	13.8
**Sex**					
Male	54.5	48.0	48.0	43.5	49.1
Female	45.5	52.0	52.0	56.5	50.9
**Male by age group**					
18–29	39.0	10.0	13.6	7.1	28.8
30–44	36.0	26.0	33.0	22.9	15.4
45–64	21.3	49.0	42.0	48.6	42.3
65+	3.7	15.0	11.4	21.4	13.5
**Female by age group**					
18–29	29.6	12.6	29.1	12.4	19.7
30–44	41.7	26.1	20.4	20.2	28.8
45–64	25.2	45.0	35.0	34.8	34.8
65+	3.5	16.2	15.5	32.6	16.7
**Household Income**					
<$20-000	8.0	11.8	19.3	8.8	11.9
$20,000-<$40,000	20.5	19.3	25.5	25.7	22.5
$40,000-<$60,000	18.8	22.5	19.9	27.9	21.8
$60,000-<$80,000	15.6	17.6	10.6	9.6	13.7
$80,000+	37.1	28.9	24.8	27.9	30.2
**Educational attainment**					
<High-school	33.9	34.3	29.0	31.2	32.3
College	28.6	26.3	36.1	22.7	28.6
University/Post-grad	37.4	39.4	35.0	46.1	39.1
**Residence status**					
Rural	16.5	16.0	18.7	19.0	17.5
Urban	33.5	84.0	81.3	81.0	82.5
**Parenthood**					
Children <6 years of age	64.5	29.4	37.7	39.7	41.8
No children <6 years of age	36.0	70.6	62.3	60.3	58.2
**Private health insurance coverage**					
Insured	79.5	65.5	43.8	54.0	66.9
Non-insured	20.5	34.5	56.2	46.0	33.1

Bivariately, young adults (18–29 years) are more likely than those 65 years or older to prefer new government spending on child care (OR=11.4, CI:5.48-23.7, *p*=.000) and with increasing age, adults are less likely to consider child care a priority (Table 2). Preference for new spending is almost cut in half among very low income families (<$20,000) compared to individuals in the highest income category (OR=0.48, CI:0.27-0.85, *p*=.012). These associations do not remain significant when all predictive variables are included in the adjusted model. In the adjusted model, only parenthood and sex remain predictive; the effect of having one or more children less than 6 years of age is associated with an increased preference (OR=4.73, CI:2.56-8.75, *p*=.000), and males are 2.2 times more likely to prefer new spending than females (CI: 1.25-3.79, *p*=.006).

**Table 2:** Results of logistic regression analysis for the odds of ranking child care first for new government spending (No=0, Yes=1)

**Characteristics**	**Model 1: Unadjusted OR**†		**Model 2: Adjusted OR**‡	
	**Odds ratio [95% CI]**	***P***	**Odds ratio [95% CI]**	***P***
**Sex**				
Male	1.40 [1.05,1.86]	.022	2.18 [1.25,3.79]	.006
Female (reference)				
**Age**				
18**–**29	11.4 [5.48,23.7]	.000	1.65 [.11,24.2]	.715
30–44	8.12 [3.95,16.7]	.000	1.05 [.08,14.4]	.968
45–64	2.88 [1.38,5.98]	.005	.65 [.05,9.13]	.749
65+ (reference)				
**Household income**				
<$20-000	.48 [.27,.85]	.012	.92 [.30,2.84]	.889
$20,000-<$40,000	.69 [.45,1.05]	.085	.72 [.28,1.85]	.501
$40,000-<$60,000	.66 [.43,1.02]	.061	.67 [.31,1.46]	.313
$60,000-<$80,000	.92 [.57,1.49]	.743	.91 [.40,2.06]	.823
$80,000+ (reference)				
**Educational attainment**				
<High-school	1.10 [.77,1.57]	.593	1.75 [.82,3.74]	.151
College	1.08 [.74,1.56]	.704	1.16 [.58,2.32]	.672
University/Post-grad (reference)				
**Parenthood**				
Children <6 years of age	4.21[2.68,6.63]	.000	4.73 [2.56,8.75]	.000
No children <6 years of age reference				
**Residence status**				
Rural	.97 [.66, 1.42]	.867		
Urban (reference)				

†Model 1 entered all variables independently.

‡ Model 2 entered variables significant (p<0.05) deemed important from model 1.

Bivariately, age is the only variable that predicts a preference for new government spending on pharmacare (Table 3). With increased age, adults are more likely to prefer new government spending. Seniors are 3.3 (Inverted OR=3.29, CI: 1.61-6.71, *p*=.001) times more likely to prefer new spending than young adults.

**Table 3:** Results of logistic regression analysis for the odds of ranking pharmacare first for new government spending (No=0, Yes=1)

**Characteristics**	**Model 1: Unadjusted OR**†		**Model 2: Adjusted OR**‡	
	**Odds ratio [95% CI]**	***P***	**Odds ratio [95% CI]**	***P***
**Sex**				
Male	.98 [.73, 1.32]	.905	.89 [.62,1.26]	.494
Female (reference)				
**Age**				
18**–**29	.43 [.24, .77]	.005	.30 [.15,.62]	.001
30–44	.81 [.50, 1.32]	.402	.53 [.30,.94]	.028
45–64	1.26 [.80, 1.98]	.320	.85 [.50,1.44]	.542
65+ (reference)				
**Household income**				
<$20-000	1.16 [.66,2.04]	.604	.98 [.51,1.86]	.938
$20,000-<$40,000	.92 [.57,1.47]	.713	.91 [.52,1.56]	.719
$40,000-<$60,000	1.16 [.73, 1.84]	.518	.99 [.59,1.66]	.989
$60,000-<$80,000	1.52 [.92, 2.52]	.106	1.64 [.96,2.81]	.071
$80,000+ (reference)				
**Educational attainment**				
<High-school	1.02 [.71, 1.46]		1.03 [.66,1.59]	.897
College	.90 [.69, 1.32]	.583	.933 [.60,1.46]	.763
University/Post-grad (reference)				
**Residence status**				
Rural	.92 [.61, 1.37]	.671	[Not included in model]	
Urban (reference)				

† Model 1 entered all variables independently.

‡ Model 2 entered variables significant (p<0.05) deemed important from model 1.

For dental care, sociodemographic variables at the bivariate level (household income, education and private health insurance status) persisted as significant predictors when the effect of all other variables was controlled (Table 4). Preference for new spending was highest among the lowest income category (OR=2.15, CI=1.10-4.17, *p*=.024). Individuals with a college education preferred new government spending significantly more compared to individuals with higher levels of education (university/post-graduate) (OR=1.64, CI=1.02-2.65, *p*=.041). Preference for new spending was also 3 times higher among the uninsured compared to the insured (Inverted OR=3.19, CI: 2.01-4.84, p=.000). Age, which had not been significant in the bivariate level, was found to be significant in the adjusted model.

**Table 4:** Results of logistic regression analysis for the odds of ranking dental care first for new government spending (No=0, Yes=1)

**Characteristics**	**Model 1: Unadjusted OR**†		**Model 2: Adjusted OR**‡	
	**Odds ratio [95% CI]**	***P***	**Odds ratio [95% CI]**	***P***
**Sex**				
Male	0.98 [0.73,1.34]	0.933	0.99 [0.67,1.45]	0.989
Female (reference)				
**Age**				
18**–**29	1.17 [0.68,2.02]	0.579	1.52 [0.74,3.15]	0.258
30–44	0.99 [0.59,1.69]	0.988	2.03 [1.04,3.97]	0.038
45–64	1.14 [0.69,1.87]	0.619	1.88 [0.99,3.55]	0.053
65+ (reference)				
**Household income**				
<$20-000	2.70 [1.56,4.66]	0.000	2.15 [1.10,4.17]	0.024
$20,000-<$40,000	1.58 [0.97,2.56]	0.066	1.09 [0.60, 1.97]	0.787
$40,000-<$60,000	1.23 [0.74,2.05]	0.424	1.04 [0.59, 1.83]	0.893
$60,000-<$80,000	0.98 [0.53,1.81]	0.955	1.01 [0.52, 1.94]	0.977
$80,000+ (reference)				
**Educational attainment**				
<High-school	0.98 [0.65,1.46]	0.901	0.94 [0.57,1.53]	0.790
College	1.58 [1.07,2.32]	0.022	1.64 [1.02,2.65]	0.041
University/Post-grad (reference)				
**Private health insurance status**				
Insured	0.38 [0.27,0.51]	0.000	0.313 [0.20,0.48]	0.000
Non-insured (reference)				
**Residence status**				
Rural	1.19 [0.80,1.77]	0.389		
Urban (reference)				

† Model 1 entered all variables independently.

‡ Model 2 entered variables significant (p<0.05) deemed important from model 1.

Both the bivariate and the multivariable results show age and education predict a preference for new spending on home care (Table 5). Preference for new spending was significantly cut in half among those with a college education or less in comparison to those who report higher levels of education (university/post-graduate). Preference for new spending also increased with age. In the multivariate model, it also appears that individuals in the $40,000-$59,999 income category favored new government spending significantly more than individuals in the $80,000+ category (OR=1.86, CI=1.08-3.22, *p*=.026).

**Table 5:** Results of logistic regression analysis for the odds of ranking home care first for new government spending (No=0, Yes=1)

**Characteristics**	**Model 1: Unadjusted OR†**		**Model 2: Adjusted OR‡**	
	**Odds ratio [95% CI]**	***P***	**Odds ratio [95% CI]**	***P***
**Sex**				
Male	.791 [.57, 1.11]	.169	.93 [.62,1.39]	.71
Female (reference)				
**Age**				
18**–**29	.20 [.11, .68]	.000	.21 [.09,.46]	.000
30–44	.31 [.19, .51]	.000	.35 [.19,.65]	.001
45–64	.49 [.32, .77]	.002	.56 [.32,.98]	.041
65+ (reference)				
**Household income**				
<$20-000	.87 [.44, 1.74]	.693	.87 [.39,1.91]	.721
$20,000-<$40,000	1.36 [.82, 2.25]	.237	1.36 [.74.2.50]	.321
$40,000-<$60,000	1.60 [.97, 2.64]	.064	1.86 [1.08.3.22]	.026
$60,000-<$80,000	.73 [.37, 1.44]	.365	.71 [ .33,.1.51]	.374
$80,000+ (reference)				
**Educational attainment**				
<High-school	.76 [.51, 1.14]	.184	.60 [.36,.98]	.041
College	.63 [.41, .98]	.041	.57 [.34,.97]	.036
University/Post-grad (reference)				
**Residence status**				
Rural	1.21 [.79, 1.86]	.37		
Urban (reference)				

†Model 1 entered all variables independently.

‡ Model 2 entered variables significant (p<0.05) deemed important from model 1.

## Discussion

When the sample was asked to prioritize preferences on a range of public health-related goods, top priority for new funding was accorded to child care. This is not surprising, given that a large majority (83%) of Canadians believe governments play an essential role in helping parents meet their child care needs [30]. Consensus currently exists among Canadian adults (77%) that the lack of affordable child care is a serious problem and a large majority support the creation of a universal child care program [30,31]. Acquiring high quality child care can be very costly and can impose burdens on monthly household budgets [32]. Hence, the high priority status given to child care may be due to parents having difficulty accessing this care due to its cost-prohibitive nature.

A large proportion of individuals considered pharmacare as a top priority over home care, yet in previous surveys, pharmacare ranked lower than home care [20,21]. This change could partially be a response to government policy outputs: all Canadian provinces have created some form of a publicly financed drug plan for seniors. To be sure, across various social policy domains in Canada and the United States, it has been shown that the public adjusts its preferences over time in reaction to policy changes [33,34]. It is also likely that pharmacare received a high priority ranking due to the increased out-of-pocket expenses incurred by individuals as a result of rising drug prices, the introduction of more expensive drugs into the market, and shorter hospital stays that require patients to cover the costs for prescription drugs that were previously administered in-hospital [2,35].

While our results indicate that the Canadian public prefers greater public support for child care, there still exists support for new spending across all areas assessed in this study, indicating heterogeneity among Canadian adults in this regard. It appears that this heterogeneity may be explained by differences in sociodemographic characteristics. Specifically, our results indicate that preferences for new spending were shown to be based on social position and need (e.g. parenthood and preference for child care spending, financial constraints and preference for dental care spending). This suggests that the public demonstrates a higher preference for a priority when the perceived benefits of new spending can address an unmet need. The heterogeneity of preferences for new spending may also be attributed to altruistic self-interest such that an individual takes other’s welfare into account because of the effect it has on their own [36,37]. Lastly, preferences for new spending may be purely altruistic, that is, a person values new spending due to the collective concern for the well-being of others or the population at large.

The predictability of parenthood for public preferences on new government spending on child care is consistent with previous research as well. While the higher priority preference seen among younger males for child care may be surprising, a recent survey indicates that although females (85%) show greater support for a government role in child care policy in Canada, male support is not very far off (80%) [30]. Also, younger mothers are now likely employed or receiving education or training, which makes a father’s involvement and knowledge of child rearing more prevalent [38]. The amount of time married fathers spend with children has increased [39,40]. Yet, compared with mothers, fathers continue to spend considerably less time with children and this is the case even when mothers are employed [39-42]. Thus, the doubled male support for childcare spending may be due to altruistic self-interest (social preference) such that husbands/men want their wives/women to gain wellbeing from publicly funded childcare, which in turn improves their children’s wellbeing. Notwithstanding, due to the forced-choice nature of the outcome variable, these results may not fully reflect actual attitudes.

Another important finding is the predictive capacity of household income for public preference on home care spending. Preferences for new government spending towards home care were much higher among households with middle incomes ($40,000-$60,000) than comparable counterparts. This is consistent with a previous survey, which found an inverse relationship between income and the receipt of home care [43]. We postulate that middle income groups potentially have a high representation in the insurance coverage gap, whereby their income is too high to qualify for public subsidies, but not high enough to afford employer-based or privately purchased health insurance. As a result, middle income individuals rely heavily on out-of-pocket spending, leaving them the most vulnerable to having their home care needs unfulfilled [44].

Our results also demonstrate that seniors accord high priority status to new government spending for pharmacare. Older adults are more likely to have chronic health conditions than any other age group and consequently prescription drug utilization is high and more regular [45]. As mentioned, while Canadian provinces have created public drug plans, this coverage may not be adequate [2,35].

Socio demographic differences appear to account for variations in public preferences for new government spending on dental care as well. Individuals in the lowest income group, with lower levels of education and with no health (dental) insurance coverage demonstrated the highest preference. It is understandable how this invokes support for dental care spending, as income and insurance status are the dominant predictors of dental care utilization [9,46]. Additionally, age was predictive of new dental care spending. In particular middle-aged adults (30–44 years of age) were more likely to assign a high priority. This may be attributed to the additional costs to the household budget of supporting the dental needs of a child. Additional co-payments, higher premiums and out-of-pocket payments due to limitations in dental plans (such as for orthodontic treatment) all impose a financial strain. This problem is further compounded for low-income families who struggle between providing basic necessities and expensive dental care for their children [9,46].

A limitation of the present study is its cross sectional design as caution must be exercised in making causal inferences in the observed associations. Another important limitation that arises from this work is the use of telephone interview surveys. This survey has likely under-represented the portion of the population who do not have access to conventional landlines, such as disadvantaged groups and individuals who have opted to solely use cellular telephones. There is evidence that those with the lowest levels of household income are more likely to opt for cellular telephones [47]. Hence, our sample may be relatively over-represented by individuals in the upper socioeconomic categories. Additionally, fewer adults of working age are at home during the day answering landlines. As such, the sample is skewed to the left with respect to age; yet our survey data was weighted to be representative of Canadian adults, thereby compensating for this.

In addition, the outcome variables were based on a forced-choice question necessitating respondents to choose one of five spending categories. Also, there may be other priority areas not addressed in the question such as new government spending for complementary and alternative medicine or a desire to see no new spending. Furthermore, respondents did not have the opportunity to provide other priority areas that they feel may be important for new government spending. Moreover, respondents were not asked to rank these priority areas in relation to existing areas of government spending. As such, although someone may rank pharma care as the top priority out of the five priority areas, they may still rank pharma care lower than any currently funded government area. As a result, public responses may not fully reflect actual attitudes.

In surveys with low response rates, non response bias is a major concern. Although the response rate in this study was low, it is typical of the response for random digital dialing (RDD) telephone interview surveys of this nature. A recent paper from the United States has outlined the risks associated with falling response rates from RDD surveys, but the authors’ research into “non response error in telephone surveys that focus on social and political topics” and recent meta-analyses suggest that “within the limits of the experimental conditions, non response did not introduce substantial biases into the estimates” of the surveys explored [48].

Overall, our findings point to the relative importance of socio demographic characteristics in shaping preferences towards new government spending. Any attempt to develop or modify existing public policy should consider an assessment of public preferences, thus aiding in identifying specific populations to which social support programs could be targeted. It is noteworthy to mention that these spending preferences are not just a matter of an insatiable demand for general budgetary expansion, but rather, it reflects the values of the citizens for which the public system is designed and a specific need for a re-examination of funding in particular areas of public health and social policy. Previous research has shown that the public notices and responds to budgetary policy change, and over time, adjusts its preferences for “less” spending when resource allocation in a priority area increases and is sufficiently meeting needs [33]. Hence, public preferences are in a sense signals to governments for support of needed public health-related goods. It is also important to recognize that these signals are not being sent by a limited residual group of individuals. These spending preferences are being expressed by Canadians who may not be receiving insurance coverage as an employment benefit and whose income is too high to qualify for public assistance.

In closing, policy leaders should consider prioritization of public preferences as intrinsic to the government priority-setting process. Citizen engagement in the policy-making process not only ensures that public health-related goods are distributed in a manner that best suits population need but also attempts to enhance public confidence in the public health sector by improving accountability. Thus, public preferences should be incorporated in a policy-setting approach, in order to ensure that public needs are met fairly and judiciously under reasonable resource constraints.

## Competing interests

The authors declare that they have no competing interests.

## Author’s contributions

SR performed statistical analyses, interpretation of the findings, wrote and prepared the manuscript. CQ conceived of the study, provided the data, edited and contributed to the revision of the manuscript. All authors read and approved the final manuscript.
